# A systematic review of the clinical impact of small colony variants in patients with cystic fibrosis

**DOI:** 10.1186/s12890-023-02611-4

**Published:** 2023-09-01

**Authors:** Harrigan Ryan, Emma Ballard, Rebecca E. Stockwell, Christine Duplancic, Rachel M. Thomson, Kimberley Smith, Scott C. Bell

**Affiliations:** 1 Centre for Children’s Health Research, Faculty of Medicine, The University of Queensland, South Brisbane, QLD Australia; 2 Statistics Unit, QIMR Berghofer Medical Research Institute, Herston, QLD Australia; 3 Adult Cystic Fibrosis Centre, The Prince Charles Hospital, Chermside, QLD Australia; 4 Respiratory Research Group, Gallipoli Medical Research Foundation, Greenslopes, QLD Australia; 5 Translational Research Institute, Woolloongabba, QLD Australia

**Keywords:** Small colony variants, Cystic fibrosis, Prevalence, Clinical impact

## Abstract

**Background:**

Cystic fibrosis (CF) is a life-limiting disorder that is characterised by respiratory tract inflammation that is mediated by a range of microbial pathogens. Small colony variants (SCVs) of common respiratory pathogens are being increasingly recognised in CF. The aim of this systematic review is to investigate the prevalence of SCVs, clinical characteristics and health outcomes for patients with CF, and laboratory diagnostic features of SCVs compared to non-small colony variants (NCVs) for a range of Gram-positive and Gram-negative respiratory pathogens.

**Methods:**

A literature search was conducted (PubMed, Web of Science, Embase and Scopus) in April 2020 to identify articles of interest. Data pertaining to demographic characteristics of participants, diagnostic criteria of SCVs, SCV prevalence and impact on lung function were extracted from included studies for analysis.

**Results:**

Twenty-five of 673 studies were included in the systematic review. Individuals infected with SCVs of *Staphylococcus aureus* (*S. aureus*) were more likely to have had prior use of the broad-spectrum antibiotic trimethoprim sulfamethoxazole (*p* < 0.001), and the prevalence of SCVs in patients infected with *S. aureus* was estimated to be 19.3% (95% CI: 13.5% to 25.9%). Additionally, patients infected with SCVs of Gram-negative and Gram-positive pathogens were identified to have a lower forced expiratory volume in one second percentage predicted (-16.8, 95% CI: -23.2 to -10.4) than those infected by NCVs. Gram-positive SCVs were commonly described as small and non-haemolytic, grown on Mannitol salt or blood agar for 24 h at 35°C and confirmed using tube coagulase testing.

**Conclusion:**

The findings of this systematic review demonstrate that SCVs of *S. aureus* have a high prevalence in the CF community, and that the occurrence of SCVs in Gram-positive and Gram-negative pathogens is linked to poorer respiratory function. Further investigation is necessary to determine the effect of infection by SCVs on the CF population.

**Supplementary Information:**

The online version contains supplementary material available at 10.1186/s12890-023-02611-4.

## Background

Cystic fibrosis (CF) is an autosomal recessive disorder leading to reduced mucociliary clearance and airway surface dehydration, resulting in impaired lung defence, chronic inflammation and infection [1–3]. Chronic bacterial infection contributes to lung function decline and requires life-long antibiotic use in people with CF [4–6]. Antibiotic exposure has been linked to the development of bacterial antibiotic resistance and other phenotypic adaptations, such as mucoidy and auxotrophic defects [7, 8]. Another emerging adaptive phenotype is that of small colony variants (SCVs).

SCVs are defined as a slow-growing bacterial subpopulation that have distinctive phenotypic traits, including small colony size due to metabolic changes, hypopigmentation and reduced virulence potential [9–13]. SCVs demonstrate higher rates of chronic infection than non-small colony variants (NCVs) in patients with CF and have higher levels of antibiotic resistance, which may contribute to ineffective clearance during treatment [9, 14–16]. The reduction of virulence factors in SCVs dampens the host-induced immune response [10, 17, 18], which enables immune system evasion in conjunction with the ability to invade host cells and persist intracellularly [19–22]. Chronic infection in CF is linked to respiratory decline and studies have highlighted that the occurrence of SCVs is associated with worsened lung function [23–25].

To date, studies of SCVs have largely focused on two of the dominant bacterial pathogens in people with CF, *S. aureus* and *Pseudomonas aeruginosa* (*P. aeruginosa*), with occasional reports of SCVs in other CF pathogens, such as the *Burkholderia cepacia (B. cepacia)* complex [26]. Currently, there has been no collation of the literature surrounding SCVs and their clinical impact on patients with CF.

The aim of this systematic review is to investigate the prevalence of SCVs, the clinical characteristics associated with their occurrence, including potential risk factors for SCV development and health outcomes, and the laboratory phenotypic features of SCVs to support laboratory diagnosis in patients with CF.

## Methods

A literature search was conducted in April 2020 utilising publicly available databases (PubMed, Web of Science, Embase, and Scopus). The keywords and searches used are provided in Additional file 1: Table S1-S4 and are in accordance with the Preferred Reporting Items for Systematic Reviews and Meta-Analyses (PRISMA) criteria.

### Selection criteria

The selection of studies involved two rounds of screening. Journal articles were reviewed by one author (H.R.) based on titles and abstracts. For eligible studies, full-text journal articles were assessed against the following criteria by two independent authors (H.R.; R.S.). In cases of disagreement regarding article eligibility, a third reviewer (S.B.) adjudicated.

Inclusion criteria: articles published in English; study population being patients with CF; identification of respiratory pathogens including SCVs; reporting of characteristics clinical outcomes of patients with SCVs compared with those not infected by SCVs.

Exclusion criteria: articles that were not original research articles and/or if SCV isolates were not of clinical origin (i.e. induced laboratory mutants).

### Data extraction

Studies were classified as investigating either Gram-positive or Gram-negative pathogens and were screened for data regarding prevalence, patient characteristics, health outcomes and diagnostic information. Patient characteristics and potential risk factors including age, sex, body weight, bacterial co-infections, and prior antibiotic use were extracted for both SCV and NCV cohorts. Age (median and range) at study baselines were extracted as well as age measurements from unspecified time periods within studies. Data on prior antibiotic usage included the antibiotics used and their duration of use. The prevalence of people with CF infected with SCVs was defined as the number of people who at any point had a positive SCV culture as a percentage of the patient population who were infected with that respiratory pathogen. Mean and standard deviation (SD) were extracted for the lung function measurement forced expiratory volume in one second percent predicted (FEV_1_%). Extracted laboratory diagnostic information included: the respiratory specimen type provided for culturing, SCV characterisation descriptors, agar media for culture, incubation times and conditions, and confirmation tests undertaken.

### Bias risk

The risk of bias within studies was evaluated by H.R. using a modified Newcastle–Ottawa Scale for cohort studies, cross-sectional studies, and case series studies (Additional file 1: Table S5-S7). The Joanna Briggs Institute (JBI) assessment tool was additionally applied to studies included in the prevalence meta-analysis (Additional file 1: Table S8). The risk of bias across studies was based on assessment of asymmetry in funnel plots. The risk of publication bias was investigated using the Egger test (*p*-value of ≤ 0.05).

### Statistical analysis

Analysis of demographic and diagnostic criteria data were performed using SPSS version 26.0 (IBM Corp, Armonk, NY). Categorical data was summarised as frequency and percentage and analysed using the Pearson Chi-squared test. Continuous data was checked for normality, summarised by mean and SD and examined using Student’s t-test. *P*-value of ≤ 0.05 was considered significant. Diagnostic criteria were stratified by Gram-status.

Meta-analysis were conducted for prevalence and FEV_1_% using STATASE version 16 (StatCorp, College Station, TX). A random effects model using the DerSimonian and Laird method with Freeman-Tukey double arcsine transformation was used to estimate prevalence of SCVs of Gram-positive pathogens. Where data were reported as median and range for FEV_1_%, mean and SD were calculated using the formula by Wan et al. [27]. A random effects model using the DerSimonian and Laird method was used to investigate the mean difference between FEV_1_% measures of the SCV and NCV cohorts by Gram status and overall. Sensitivity analysis was performed following completion of the meta-analysis, in which studies deemed to be substantially outside the pseudo 95% confidence limits of the funnel plot were removed to determine their impact on the results of the meta-analysis. Heterogeneity across studies was investigated using the Chi-squared test and I^2^ value. An I^2^ value for under 30% represents little heterogeneity while 75% or more represents considerable heterogeneity [28].

## Results

The literature search screening (Additional file 1: Figure S1) identified 673 records to be assessed; in the first phase of screening, 616 citations were removed, resulting in 57 full-text articles for the second phase assessment. There was only one case of reviewer disagreement, in which another author (S.B.) adjudicated the decision; 25 full-text articles were included in the systematic review. The characteristics of the included studies and their reported measures are shown in Table 1.

**Table 1:** Included studies in the systematic review

**Papers**		**Study Methodology**				**Clinical and Microbiological Data**				
**Study**	**Country**	**Study Design**	**Cohort**	**Total Participants**	**Bacterial Pathogen**	**SCV Prevalence**	**SCV Diagnosis**	**AST of SCVs**	**Lung Function Parameters**	**Risk factors for SCVs**
Anderson et al*.* [29]	USA	Cross-sectional	NA	NA	*S. maltophilia*	✓	✓	✓		
Besier et al*.* [11]	Germany	Prospective cohort	Adult and Paediatric	252	*S. aureus*	✓	✓	✓	✓	✓
Besier et al*.* [30]	Germany	Cross-sectional	NA	NA	*S. aureus*	✓	✓	✓		
Carzino et al*.* [31]	Australia	Prospective cohort	Paediatric	41	*S. aureus*	✓	✓			
De Souza et al*.* [32]	Brazil	Case series	Paediatric	1	*S. aureus*		✓	✓		
Dodement et al*.* [33]	Belgium	Prospective cohort	Adult and Paediatric	510	*S. aureus*	✓	✓	✓		✓
Haussler et al*.* [26]	Germany	Prospective cohort	Adult and Paediatric	346	*P. aeruginosa*	✓	✓	✓	✓	
Haussler et al*.* [34]	Germany	Prospective cohort	Adult and Paediatric	470	Bcc	✓	✓		✓	
Junge et al*.* [35]	Germany	Prospective cohort	Adult and Paediatric	195	*S. aureus*	✓	✓		✓	✓
Kahl et al*.* [36]	Germany	Prospective cohort	Adult and Paediatric	78	*S. aureus*	✓	✓	✓		✓
Kahl et al*.* [37]	Germany	Prospective cohort	Adult and Paediatric	72	*S. aureus*	✓				
Lozano et al*.* [38]	Spain	Case series	NA	NA	*P. aeruginosa*			✓		
Masoud-Landgraf et al*.* [25]	Austria	Cross-sectional	Adult and Paediatric	147	*S. aureus*	✓	✓	✓		
Moisan et al*.* [19]	Canada	Cross-sectional	NA	17	*S. aureus*	✓	✓	✓		
Morelli et al*.* [16]	Italy	Cross-sectional	Adult and Paediatric	222	*S. aureus*	✓	✓	✓		✓
Pakasticali et al*.* [39]	Turkey	Cross-sectional	Adult and Paediatric	84	*S. aureus*	✓	✓	✓		✓
Precit et al*.* [40]	USA	Cross-sectional	Paediatric	23	*S. aureus*		✓	✓		
Schneider et al*.* [24]	Switzerland	Cross-sectional	Adult and Paediatric	98	*S. aureus* and *P. aeruginosa*	✓	✓		✓	✓
Schwerdt et al*.* [41]	Germany	Retrospective cohort	Adult and Paediatric	283	*S. aureus*	✓			✓	
Suwantarat et al*.* [42]	USA	Cross-sectional	Adult and Paediatric	NA	*S. aureus*	✓	✓	✓	✓	✓
Tkadlec et al*.* [43]	Czech Republic	Cross-sectional	Adult and Paediatric	107	*S. aureus*	✓	✓	✓		✓
Vergison et al*.* [44]	Belgium	Prospective cohort	Adult and Paediatric	594	*S. aureus*	✓	✓	✓		✓
Wolter et al*.* [23]	USA	Prospective cohort	Paediatric	100	*S. aureus*	✓	✓		✓	✓
Wolter et al*.* [14]	USA	Prospective cohort	Paediatric	230	*S. aureus*	✓	✓		✓	✓
Yagci et al*.* [12]	Turkey	Prospective cohort	Adult and Paediatric	248	*S. aureus*	✓	✓	✓		✓

SCV small colony variant, *S. aureus Staphylococcus aureus, P. aeruginosa Pseudomonas aeruginosa*, *Bcc Burkholderia cepacia* complex, *AST* Antimicrobial susceptibility testing

Of the 25 included studies, 12 (48.0%) were of longitudinal cohort design, 10 (40.0%) were cross-sectional, 2 (8.0%) were case series, and 1 (4.0%) was a retrospective cohort study. Most studies were conducted in Europe (*n* = 15) and North America (*n* = 5); 16 (64.0%) studies included both adult and paediatric patients, while 5 (20.0%) were paediatric only and 4 (16.0%) did not describe the age of the population. Cohorts ranged from 1 to 594 patients. All the Gram-positive studies identified (*n* = 21) focused on *S. aureus*. Studies investigating Gram-negative pathogens (*n* = 5) investigated *P. aeruginosa* (*n* = 3), *Stenotrophomonas maltophilia* (*n* = 1), and *B. cepacia* complex (*n* = 1). There was one study [24] that examined both a Gram-positive and a Gram-negative pathogens.

### Within study risk of bias

All studies, except two (one *S. aureus* study [19] and one *P. aeruginosa* study [34]), were rated as being of medium to high quality of evidence using the Newcastle–Ottawa Scale. Studies (*n* = 16) included in the prevalence meta-analysis were additionally analysed using the JBI assessment tool, and all were of medium to high quality except for the same two studies identified by the Newcastle–Ottawa Scale as low quality. These low rated studies were excluded from all meta-analyses. The full risk of bias assessments is shown in Additional file 1: Tables S5-S8.

### Demographic characteristics

Patient characteristics commonly reported include age (*n* = 21, 84.0%), sex (*n* = 15, 60.0%), and prior antibiotic use (*n* = 12, 48.0%). Of these, the only factor shown to be associated with SCV occurrence was the prior use of the antibiotic trimethoprim sulfamethoxazole (SXT). Six (24.0%) studies were included in the analysis of prior antibiotic use and were all studies on SCVs of *S. aureus*; in addition to reporting for both SCV and NCV patients, these studies defined the antibiotics and time periods investigated. Two studies investigated antibiotic use in the preceding 36 months, 2 studied the preceding 12 months, 1 studied the preceding six months, and 1 studied ongoing antibiotic use. Prior use of the antibiotic SXT was more common in SCV patients (68.2%) than NCV patients (28.5%) (*p* < 0.001) (Table 1). Five (20.0%) and 7 (28.0%) studies were deemed valid for analysis of age and sex, respectively, as these studies reported SD or range for continuous variables and reported for both SCV and NCV infected cohorts. No difference was found in average median age or sex proportions between the SCV and NCV cohorts (Table 2).

**Table 2:** Demographic characteristics and risk factors associated with SCV and NCV presence

	**Valid studies (n)**	**Total number of patients**	**SCV Cohort**	**NCV Cohort**	***p*-value**
Age (Mean (SD) of median values)	4	346	16.9 (3.4)	13.0 (5.1)	0.20
Sex, males (n (%))	7	704	104 (44.8)	209 (44.3)	0.89
Prior use of SXT^a,b^ (n (%))	6	449	86 (68.2)	97 (28.5)	< 0.001

^a^Trimethoprim/sulfamethoxazole

^b^Investigation of prior use of SXT is limited to studies of *S. aureus*

Less commonly reported characteristics include bodyweight (*n* = 7, 28.0%) and co-infection with other bacterial pathogens (*n* = 10, 40.0%). Statistical analysis of differences in bodyweight and co-infection between the SCV and NCV cohorts was not conducted due to a lack of consistency in studies reporting these characteristics.

### Prevalence meta-analysis

**Fig. 1:**
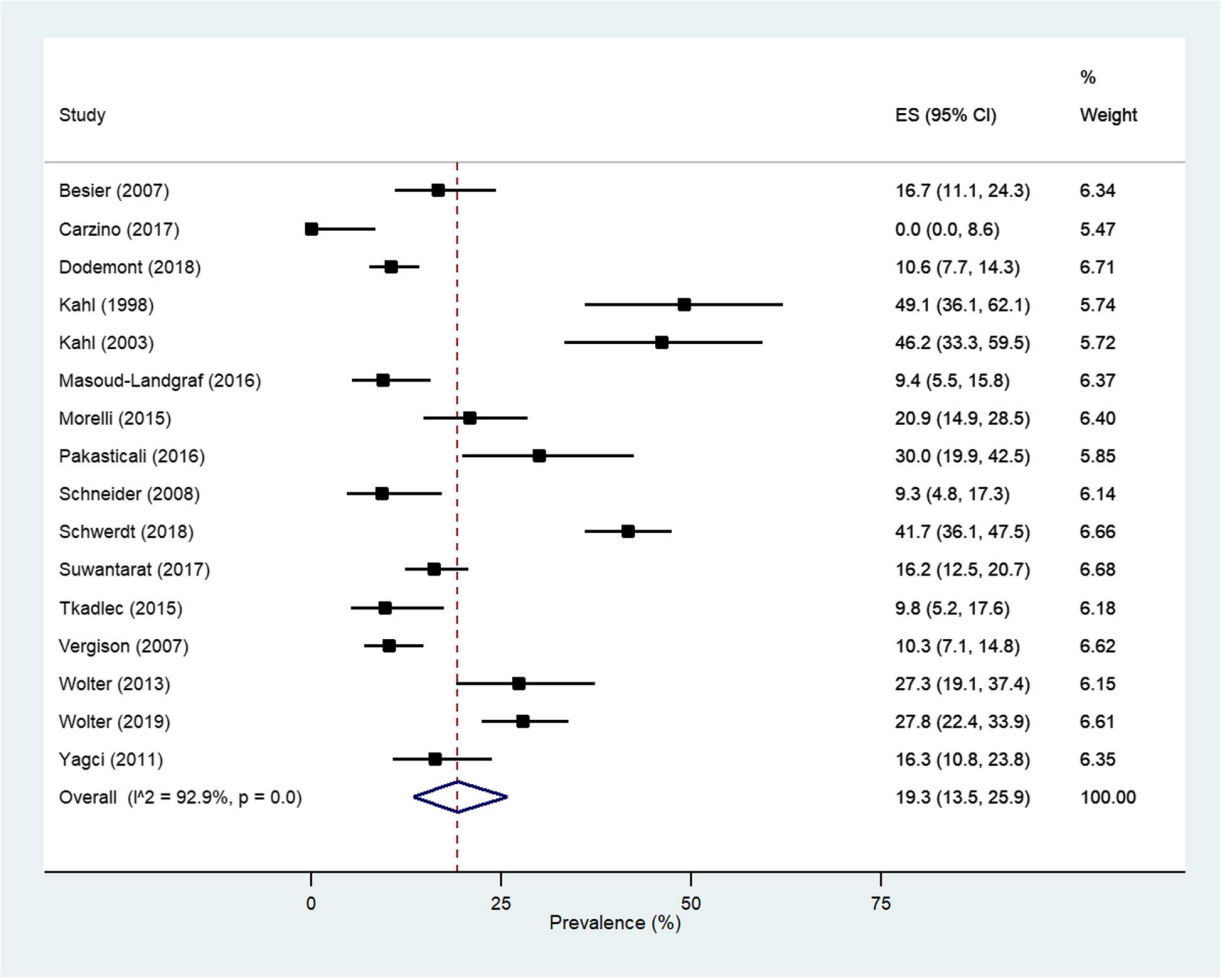
Forest plot of the reported prevalence of Gram-positive (*S. aureus*) SCVs in included studies (*n* = 16) utilising a random effects model with the DerSimonian and Laird method. Diamond represents the estimated prevalence of SCVs. ES, Estimate Size

An SCV prevalence rate of 19.3% (95% CI: 13.5 to 25.9%) was determined for *S. aureus* in a meta-analysis of 16 (64.0%) studies (Fig. 1). Gram-negative pathogens were excluded from analysis due to the small number of studies (*n* = 5) and range of pathogens being investigated.

The symmetrical distribution of studies within the pseudo 95% confidence limits of the funnel plot (Additional file 1: Figure S2) indicate a lack of small study effects, in which estimated prevalence is skewed by small studies demonstrating larger treatment effects than large studies (*p* = 0.68, Egger’s test). There is evidence of significant heterogeneity, with an I^2^ value of 92.9%, and with two studies being outside of the pseudo 95% confidence intervals. The most significant [41] was removed for sensitivity analysis and prevalence decreased to 17.9% (95% CI: 12.0—23.4%) (Additional file 1: Figures S3-S4).

### FEV_1_% meta-analysis

**Fig. 2:**
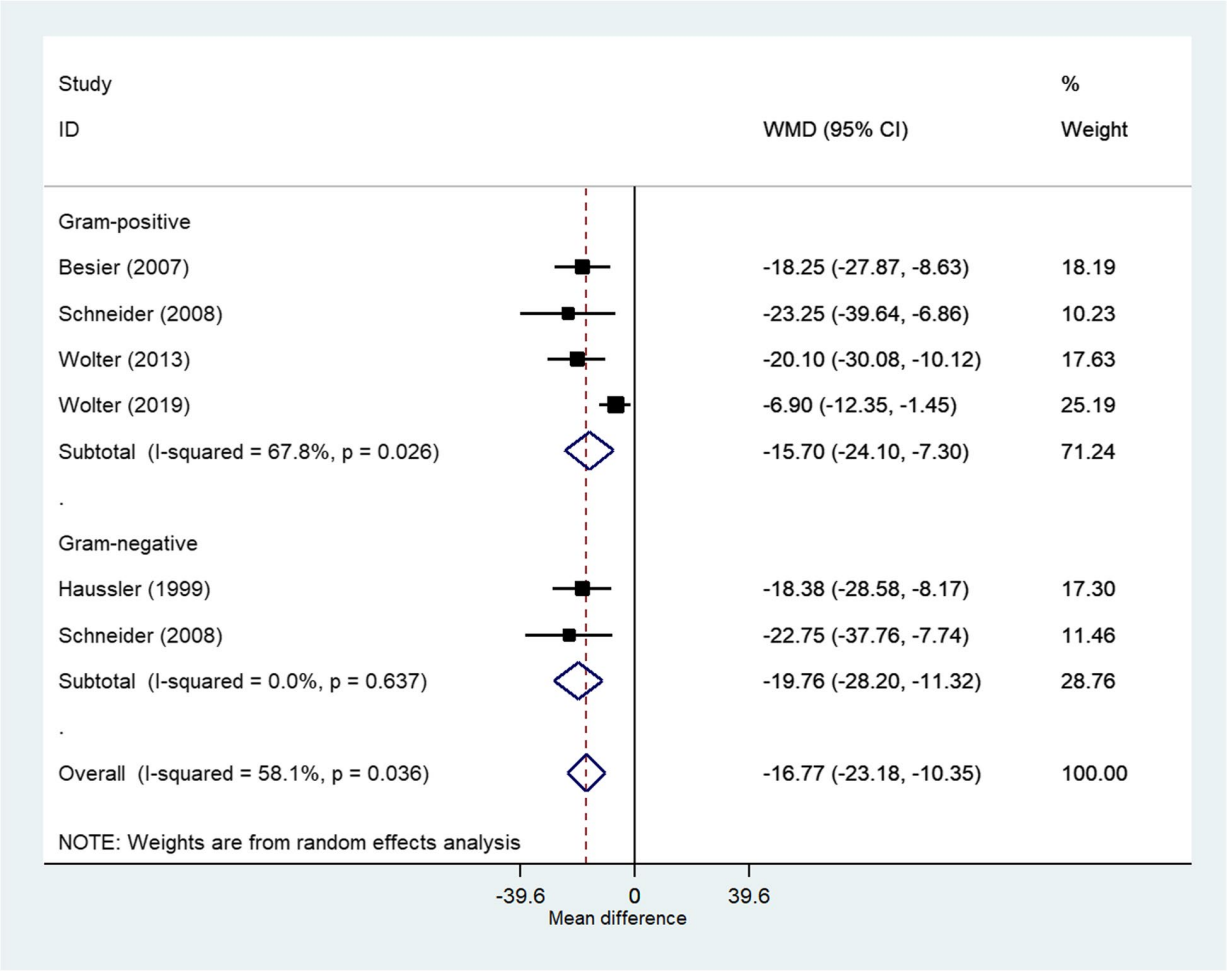
Forest plot of the reported mean difference in FEV_1_% between SCV and NCV patients in included studies (*n* = 6) utilising a random effects model with the DerSimonian and Laird method. Diamond represents weighted mean difference between SCV and NCV patients. Estimates by Gram status and overall are given

Five (20.0%) studies were included in a meta-analysis to compare the lung function of patients in the SCV and NCV cohorts. One study [28] was included in the analysis twice due to providing FEV_1_% measures for both Gram-positive and Gram-negative patients infected with SCVs. The meta-analysis showed that FEV_1_% was 16.8% lower (95% CI: -23.2 to -10.4) in patients infected with SCVs compared to patients infected with NCVs (Fig. 2).

The distribution of studies within the funnel plot was asymmetrical (Additional file 1: Figure S5), which indicates that small study effects could be present (*p* = 0.007, Egger’s test). There is some evidence of heterogeneity, with one study (Wolter et al. 2019) outside of the pseudo 95% confidence limits of the forest plot (Additional file 1: Figure S5) and an I^2^ value of 58.1% (Fig. 2).

Sensitivity analysis was performed with the removal of the outlier, Wolter et al. [14]. This reduced heterogeneity and shifted the estimated mean difference for the Gram-positive group closer to that of the Gram-negative group and increased the mean difference to -19.8% (95% CI: -24.9 to -14.7) (Additional file 1: Figure S6-S7).

### Identification of SCVs

#### Sample types

For both Gram-positive and Gram-negative pathogens, respiratory sputum samples were the most commonly acquired from patients (17/18 (94.4%) and 2/3 (66.7%) of valid studies). It was common for studies to use multiple sampling techniques, and deep throat respiratory swabs were the next most common sample acquired from patients (12/18 (66.7%) and 1/3 (33.3%)) (Additional file 1: Table S9).

#### Phenotypic characterisation

A variety of different phenotypic descriptors were described in both Gram-positive and Gram-negative studies. The most common phenotypic colony descriptions in studies of infection by Gram-positive pathogens were ‘small’ and ‘non-haemolytic’ (both 17/19(89.5%)) followed by ‘greyish/non-pigmented’ (16/19 (84.2%)) and ‘slow-growing’ (7/19 (36.8%)) (Additional file 1: Table S10). Characteristics such as ‘slow-growing’, ‘small’ and ‘maintaining small phenotype in at least two subcultures’ were reported in a limited number of Gram-negative studies (Additional file 1: Table S10).

#### Agar media

Seventeen Gram-positive studies reported the agar media used for the cultivation of SCVs. The most common were Mannitol salt agar (11/17 (64.7%)) and blood agar (10/17 (58.8%)) (Additional file 1: Table S11). Columbia sheep blood agar were reported in a limited number of Gram-negative studies (Additional file 1: Table S11).

#### Incubation time and conditions

Concerning incubation times, 11 out of 16 (68.8%) of valid Gram-positive studies utilised 24 h of incubation time compared with all valid Gram-negative studies reporting an incubation time of 48 h (Additional file 1: Table S12). Furthermore, the incubation temperature of 35°C (11/16 (68.8%)) was common for Gram-positive studies, whereas 2 out of 3 (66.7%) of valid Gram-negative studies used temperatures of 35°C or 37°C (Additional file 1: Table S12).

#### Confirmation testing

Gram-positive species were commonly confirmed using tube coagulase testing (11/18 (61.1%)), agglutination testing (10/18 (55.6%)) and PCR amplification of the *nucA* gene (8/18 (44.4%)) (Additional file 1: Table S13). Limited Gram-negative studies gave information regarding follow-up confirmation testing.

## Discussion

Chronic bacterial respiratory infection plays a primary role in the morbidity and mortality of CF [6, 45, 46], however, the contribution of SCVs of these bacterial respiratory pathogens to these adverse health outcomes is relatively unknown. This systematic review identifies that the incidence of SCVs of *S. aureus* are associated with the prior use of SXT, an antibiotic commonly used in the treatment of respiratory infections in patients with CF [47, 48], and that SCVs of *S. aureus* are highly prevalent in the CF population. Furthermore, this review found that those infected with SCVs of Gram-negative and Gram-positive pathogens were found to have lower FEV_1_% than those infected with NCVs.

Our results demonstrated prior use of SXT is associated with higher rates of SCVs of *S. aureus*. This confirms the findings of other published studies that postulate that SCVs are an adaptive phenotype to antibiotic pressure [10, 49], and that SXT use in the treatment of *S. aureus* infections has been linked to the development of SCVs [9, 47, 48].

Of the well-recognised CF bacterial respiratory pathogens, our meta-analysis identified that SCVs of *S. aureus* were highly prevalent in the CF population. *S. aureus* is one of the dominant pathogens in CF [50], especially in the younger age groups, and the high prevalence of SCVs in this species could have significant ramifications regarding management of infections. The results of this meta-analysis are robust; there is a low risk of small study effects and although heterogeneity between studies was detected, this is typical of a prevalence meta-analysis [51].

Lung function was found to be worse in patients infected with SCVs, which confirms the results of other studies which have highlighted SCV incidence to be associated with worsened lung function [23, 24]. The meta-analysis of the collated literature demonstrated that SCV patients have lower FEV_1_% than those infected with NCVs, which is a significant finding as morbidity in people with CF is often due to respiratory failure [45, 52]. The sensitivity analysis removed one study [14]; the remaining studies had a broad range of sample sizes (98 to 346 participants), and there was no significant impact on the results, indicating minimal effect of Wolter et al. [14] on the meta-analysis. However, the lower lung function may not just be a result of SCV respiratory infection, as the lowered lung function observed may also be part of CF disease progression and co-morbidities [53]. There has been no prior collation of the diagnostic methods and identifiers used to isolate SCVs in patients with CF. However, similar to the identification of other mutants of respiratory pathogens, such as mucoidy *P. aeruginosa* [8], diagnosis appears to be achieved through a combination of laboratory isolation techniques based on phenotypic characteristics, as well as the use of molecular or biochemical tests to confirm SCV presence. Phenotypic characteristics reported were diverse, but descriptors such as ‘small’ and ‘slowing-growing’ were commonly reported by both Gram-positive and Gram-negative studies, as well as descriptors ‘non-haemolytic’ and ‘greyish/non-pigmented’ being common in solely Gram-positive studies. The assessment of SCVs is not currently routine practice in the work-up of CF respiratory samples in clinical microbiology laboratories [14, 54]; long-term observational studies of the clinical outcomes of patients with SCVs are required to provide evidence of adverse impact to support outside use of a research setting.

This systematic review has several limitations. Articles included were limited to those published in English, and inferences from Gram-negative studies were difficult due to the limited number of included studies. Prior investigation into SCVs has primarily focused on *S. aureus*, and as such, there were limited Gram-negative studies available and different Gram-negative species were grouped together for analysis. Limitations of certain statistical analyses also must be considered; the FEV_1_% meta-analysis had a small number of included studies, and in three of the five studies, the means and standard deviations for analysis were acquired through the conversion of study medians, minimums and maximums as described by [27]. The analysis of diagnostic methods used only descriptive frequencies of the diagnostic methods reported, thus the results must be considered with caution. Due to an inconsistency in the reporting of BMI and antimicrobial susceptibility testing results, statistical testing was unable to be performed.

The review’s findings were primarily based on studies studying SCVs of *S. aureus*; the literature surrounding other respiratory pathogens is lacking, and further study should focus on their incidence and strengthening diagnostic methods. Additionally, although most of the literature has focused on SCVs in CF, other disease states, such as osteomyelitis and device-related infections [55–58], have reported their incidence. Study into their incidence in other disease states could elucidate how SCV prevalence varies. Enhanced understanding of SCV incidence and diagnosis would enable further studies into their impact on health outcomes. Furthermore, the recent addition of CFTR modulator therapies in patients with suitable CFTR variants (up to 90% of the CF population) has led to a significant reduction in pulmonary exacerbations, changes in airway microbiota and reduced intensity of antibiotic therapy [59, 60]. Impacts of these changes may impact the prevalence of SCV infection in patients with CF and also the long-term adverse consequences of SCVs. The changing prevalence of CF airway infections will be the subject of ongoing clinical research and provides an opportunity for SCVs in people with CF.

SCVs were found to be prevalent in people with CF and associated with the prior use of SXT. Furthermore, a trend towards individuals infected with SCVs having lower FEV_1_% than those infected with NCVs was observed. Bacterial infection in CF plays a large role in adverse health outcomes, and the further study of SCVs and how they bring about these adverse outcomes will be necessary to better the treatment and management of CF in future.

### Supplementary Information

**Additional file 1: Table S1.** PubMed search strategy.** Table S2.** Web of Science search strategy. **Table S3.** Embase search strategy. **Table S4.** Scopus search strategy. **Table S5.** Bias assessment of cohort studies. **Table S6.** Bias assessment of cross-sectional studies. **Table S7.** Bias assessment of case series studies. **Table S8.** Bias assessment for prevalence studies**. Figure S1.** Flow diagram of search procedure. **Figure S2.** Funnel plot for prevalence of SCVs. **Figure S3.** Sensitivity analysis forest plot for prevalence of SCVs. **Figure S4.** Sensitivity analysis funnel plot of prevalence of SCVs. **Figure S5.** Funnel plot for mean difference of FEV_1_% between SCV and NCV participants. **Figure S6.** Sensitivity analysis forest plot for mean difference of FEV_1_% between SCV and NCV participants. **Figure S7.** Sensitivity analysis funnel plot for the mean difference of FEV_1_% between SCV and NCV participants. **Table S9.** Specimens used for SCV collection. **Table S10.** Growth characteristics of SCVs. **Table S11.** Agar mediums for SCV cultivation. **Table S12.** Incubation conditions for SCVs. **Table S13.** Tests used for SCV confirmation.

## Data Availability

The datasets used and/or analysed during the current study are available from the corresponding author on reasonable request.

## Abbreviations

AST: Anti-microbial susceptibility testing
CF: Cystic fibrosis
FEV_1_%: Forced expiratory volume in one second percentage predicted
NCV: Non-small colony variant
*P. aeruginosa*: *Pseudomonas aeruginosa*
*S. aureus*: *Staphylococcus aureus*
SCV: Small colony variant
SD: Standard deviation
SXT: Trimethoprim sulfamethoxazole
