# Supplementary material for: A systematic review of the clinical impact of small colony variants in patients with cystic fibrosis
Source: BMC Pulm Med. 2023 Sep 1;23:323. doi: 10.1186/s12890-023-02611-4 (PMC10474644; doi:10.1186/s12890-023-02611-4)
Supplement: Supplementary file 1 — Additional file 1: Table S1. PubMed search strategy. Table S2. Web of Science search strategy. Table S3. Embase search strategy. Table S4. Scopus search strategy. Table S5. Bias assessment of cohort studies. Table S6. Bias assessment of cross-sectional studies. Table S7. Bias assessment of case series studies. Table S8. Bias assessment for prevalence studies. Figure S1. Flow diagram of search procedure. Figure S2. Funnel plot for prevalence of SCVs. Figure S3. Sensitivity analysis forest plot for prevalence of SCVs. Figure S4. Sensitivity analysis funnel plot of prevalence of SCVs. Figure S5. Funnel plot for mean difference of FEV1% between SCV and NCV participants. Figure S6. Sensitivity analysis forest plot for mean difference of FEV1% between SCV and NCV participants. Figure S7. Sensitivity analysis funnel plot for the mean difference of FEV1% between SCV and NCV participants. Table S9. Specimens used for SCV collection. Table S10. Growth characteristics of SCVs. Table S11. Agar mediums for SCV cultivation. Table S12. Incubation conditions for SCVs. Table S13. Tests used for SCV confirmation. [file 12890_2023_2611_MOESM1_ESM.docx]

**Table S1: Search strategy and search terms of PubMed**

| Database | PubMed | | | |
| --- | --- | --- | --- | --- |
| Search Number | **Search** | **Number of Search Results** | **References Exported?** | **Date Searched** |
| 1 | “Cystic fibrosis” [TIAB] | 43869 | No | 06/04/20 |
| 2 | “Cystic fibrosis” [MeSH Terms] | 34782 | No | 06/04/20 |
| 3 | “CF” [TIAB] | 39929 | No | 06/04/20 |
| 4 | “Lung diseases” [MeSH] | 870968 | No | 06/04/20 |
| 5 | “lung disease” [TIAB] | 40000 | No | 06/04/20 |
| 6 | “Cystic fibrosis” [TIAB] OR “cystic fibrosis” [MeSH Terms] OR “CF” [TIAB] OR “Lung diseases” [MeSH] OR “lung disease” [TIAB] | 917527 | No | 06/04/20 |
| 7 | “Small colony variant*” [TIAB] | 225 | No | 06/04/20 |
| 8 | “SCV*” [TIAB] | 1111 | No | 06/04/20 |
| 9 | “Persister cell*” [TIAB] | 115 | No | 06/04/20 |
| 10 | “Small colony variant*” [TIAB] OR “SCV*” [TIAB] OR “persister cell*” [TIAB] | 1312 | No | 06/04/20 |
| 11 | (“Small colony variant*” [TIAB] OR “SCV*” [TIAB] OR “persister cell*” [TIAB]) AND (“cystic fibrosis” [MeSH Terms] OR “Cystic fibrosis” [TIAB] OR “CF” [TIAB] OR “Lung diseases” [MeSH] OR “lung disease” [TIAB]) | 78 | Yes | 06/04/20 |

**Table S2: Search strategy and search terms of Web of Science**

| Database | Web of Science | | | |
| --- | --- | --- | --- | --- |
| Search Number | Search | Number of Search Results | References Exported? | Date Searched |
| 1 | “Cystic fibrosis” | 68701 | No | 06/04/20 |
| 2 | CF | 77187 | No | 06/04/20 |
| 3 | “Lung disease” | 50893 | No | 06/04/20 |
| 4 | “Cystic fibrosis” OR CF OR “lung disease” | 174115 | No | 06/04/20 |
| 5 | “Small colony variant” | 276 | No | 06/04/20 |
| 6 | “Small colony variants” | 913 | No | 06/04/20 |
| 7 | SCV* | 2662 | No | 06/04/20 |
|  | “Persister cell” | 133 | No | 06/04/20 |
|  | “Persister cells” | 809 | No | 06/04/20 |
|  | “Small colony variant” OR “small colony variants” OR CF “persister cell” OR “persister cells” | 1819 | No | 06/04/20 |
| 8 | “cystic fibrosis” OR CF OR “lung disease”) AND (“small colony variant” OR “small colony variants” OR SCV* OR “persister cell” OR “persister cells”) | 388 | Yes | 06/04/20 |

**Table S3: Search strategy and search terms of Embase**

| Database | Embase | | | |
| --- | --- | --- | --- | --- |
| Search Number | Search | Number of Search Results | References Exported? | Date Searched |
| 1 | ‘Cystic fibrosis’:ti,ab | 65556 | No | 06/04/20 |
| 2 | ‘cystic fibrosis’/exp | 72212 | No | 06/04/20 |
| 3 | ‘CF’:ti,ab | 69804 | No | 06/04/20 |
| 4 | ‘Lung disease’/exp | 1477833 | No | 06/04/20 |
| 5 | ‘Lung disease’:ti,ab | 65542 | No | 06/04/20 |
| 6 | #1 OR #2 OR #3 OR #4 OR #5 | 1579705 | No | 06/04/20 |
| 7 | ‘Small colony variant*’:ti,ab | 645 | No | 06/04/20 |
| 8 | ‘SCV*’:ti,ab | 3097 | No | 06/04/20 |
| 9 | ‘Persister cell*’:ti,ab | 542 | No | 06/04/20 |
| 10 | #7 OR #8 OR #9 | 3876 | No | 06/04/20 |
| 11 | (‘cystic fibrosis’/exp OR ‘cystic fibrosis’:ti,ab OR ‘cf’:ti,ab OR ‘lung disease’/exp OR ‘lung disease’:ti,ab) AND (‘small colony variant*’:ti,ab OR ‘SCV*’:ti,ab OR ‘persister cell*’:ti,ab) | 410 | No | 06/04/20 |
| 12 | (‘cystic fibrosis’/exp OR ‘cystic fibrosis’:ti,ab OR ‘cf’:ti,ab OR ‘lung disease’/exp OR ‘lung disease’:ti,ab) AND (‘small colony variant*’:ti,ab OR ‘SCV*’:ti,ab OR ‘persister cell*’:ti,ab) AND [embase]/lim AND [english]/lim | 365 | Yes | 06/04/20 |

**Table S4: Search strategy and search terms of Scopus**

| Database | Scopus | | | |
| --- | --- | --- | --- | --- |
| Search Number | Search | Number of Search Results | References Exported? | Date Searched |
| 1 | “cystic fibrosis” | 66581 | No | 03/04/20 |
| 2 | “CF” | 97366 | No | 03/04/20 |
| 3 | “lung disease” | 248887 | No | 03/04/20 |
| 4 | “cystic fibrosis” OR “CF” OR “lung disease” | 389554 | No | 03/04/20 |
| 5 | “small colony variant*” | 578 | No | 03/04/20 |
| 6 | “SCV*” | 3585 | No | 03/04/20 |
| 7 | “persister cell*” | 570 | No | 03/04/20 |
| 8 | "small colony variant*" OR "SCV*" OR "persister cell*" | 4392 | No | 03/04/20 |
| 9 | (“cystic fibrosis” OR “CF” OR “lung disease”) AND ("small colony variant*" OR "SCV*" OR "persister cell*") | 153 | Yes | 03/04/20 |

**Table S5: Modified Newcastle-Ottawa Scale for Assessment of Bias in Cohort Studies (n=13)**

| Paper | | Selection | | | | Comparability | Outcome | | |  |  |
| --- | --- | --- | --- | --- | --- | --- | --- | --- | --- | --- | --- |
| Study | **Year** | **Representativeness** | **Selection of non-exposed cohort** | **Ascertainment of exposure** | **Demonstration that outcome of interest was not present at start of study** | **Comparability of cohort on the basis of design** | **Assessment of outcome** | **Sufficient follow-up** | **Adequacy of follow-up of cohorts** | **Score** | **Rating** |
| Besier *et al.* | 2007 | ** | * | * | * | * | * | * | * | 9 | High |
| Carzino *et al.* | 2017 | * | * | * | * | * | * |  |  | 6 | Medium |
| Dodemont *et al.* | 2019 | ** | * | * | * | * |  |  |  | 6 | Medium |
| Haussler *et al.* | 2003 | ** | * | * | * | * |  | * | * | 8 | High |
| Haussler *et al.* | 2003 |  | * | * | * | * |  |  |  | 4 | Low |
| Junge *et al.* | 2016 | * | * | * | * | * | * | * | * | 8 | High |
| Kahl *et al.* | 1998 | * | * | * | * | * | * |  | * | 7 | Medium |
| Kahl *et al.* | 2003 | * | * | * | * | * | * | * | * | 8 | High |
| Schwerdt *et al.* | 2018 | ** | * | * |  | * | * | * | * | 8 | High |
| Vergison *et al.* | 2007 | * | * | * | * | * | * |  |  | 6 | Medium |
| Wolter *et al.* | 2013 | * | * | * | * | ** | * | * | * | 9 | High |
| Wolter *et al.* | 2019 | * | * | * | * | ** | * | * | * | 9 | High |
| Yagci *et al.* | 2011 | ** | * | * | * | * | * |  | * | 8 | High |

Selection: Maximum of one star for each criteria

1. Representativeness 🡪 Two stars given if the patient cohort is truly representative of the average CF patient. One star given in there are no age/sex/ethnicity restrictions, and another star given if there are no exclusions of patients depending on their colonization status, i.e. patients are excluded if they are colonized with *Pseudomonas aeruginosa*
2. Selection of non-exposed cohort 🡪 One star given if the non-exposed (i.e. NCV) cohort was drawn from the same group as the SCV group
3. Ascertainment of exposure 🡪 One star given if methods of SCV identification are sufficiently described
4. Demonstration that outcome of interest was not present at the start of the study 🡪 One star given if patients are accepted in a blinded fashion, i.e. it is unknown whether or not they have SCVs prior to the start of the study

Comparability: Maximum of two stars

1. Comparability of cohort on the basis of design:

- One star given if all patients across cohorts are patients with CF
- Second star is given if the study controls for the presence of other co-morbidities that could skew the results

Outcome: Maximum of one star for each criteria

1. Assessment of outcome 🡪 One star given if measurement procedures are sufficiently described and are not self-reported by patients
2. Sufficient follow-up 🡪 One star given if each patient is tracked for one year or more
3. Adequacy of follow-up of cohorts 🡪 One star given if complete follow-up with all patients or subjects lost to follow-up unlikely to introduce bias (under 10% lost)

Rating of quality of evidence

8-10 = high quality evidence

5-7 = medium quality evidence

0-4 = low quality evidence

**Table S6: Modified Newcastle-Ottawa Scale for Assessment of Risk of Bias of Cross-sectional studies (n=10)**

|  |  | Selection | | | | Comparability | Outcomes | |  |  |
| --- | --- | --- | --- | --- | --- | --- | --- | --- | --- | --- |
| Study | **Year** | **Representativeness** | **Sample size** | **Non-respondents** | **Ascertainment of exposure** | **Comparability of cohort on the basis of design** | **Assessment of outcome** | **Statistical test** | **Score** | **Rating** |
| Anderson *et al.* | 2007 |  |  |  | * | * | * | * | 4 | Medium |
| Besier *et al.* | 2008 |  |  | * | * | * | * | * | 5 | Medium |
| Masoud-Landgraf *et al.* | 2016 | ** | * | * | * | * | * | * | 8 | High |
| Moisan *et al.* | 2005 |  |  |  | * |  | * | * | 3 | Low |
| Morelli *et al.* | 2015 | * | * | * | * | * | * | * | 7 | High |
| Pakasticali *et al.* | 2016 | * | * | * | * | * | * | * | 7 | High |
| Precit *et al.* | 2016 | * | * |  | * |  | * | * | 5 | Medium |
| Schneider *et al.* | 2008 | ** | * | * | * | * | * | * | 8 | High |
| Suwantarat *et al.* | 2018 | * | * | * | * | * | * | * | 7 | High |
| Tkadlec *et al.* | 2015 | ** | * | * | * | * | * | * | 8 | High |

Selection: Maximum of five stars

1. Representativeness of sample 🡪 Is the general population of the study representative of the average CF patient? One star given if population descriptions reveal no disparities regarding age/sex/ethnicity, and another star if there are no restrictions on what bacterial pathogens they were colonized with.
2. Sample size 🡪 given if justified and satisfactory (n>30)
3. Selection of non-exposed cohort 🡪 One star given if the non-exposed (i.e. NCV) cohort was drawn from the same group as the SCV group
4. Ascertainment of exposure 🡪 one star given if validation methods are described sufficiently

Comparability: Maximum of one star

1. Comparability on cohort on the basis of design:

- One star given if all patients across cohorts are CF patients

Outcome: Maximum of one star for each criteria

1. Assessment of outcome 🡪 One star given if assessment methods are described and are not reported by patients
2. Statistical test 🡪 if it is clearly described, appropriate and complete

Rating of quality of evidence:

High = 7-8

Medium = 4-6

Low = 0-3

**Table S7: Modified Newcastle-Ottawa Scale for Assessment of Bias Risk in Case series studies (n=2)**

|  |  | Selection | | | Comparability | Outcomes | | |  |  |
| --- | --- | --- | --- | --- | --- | --- | --- | --- | --- | --- |
| Study | **Year** | **Case description** | **Sample size** | **Ascertainment of exposure** | **Comparability** | **Defined Outcomes** | **Sufficient follow-up** | **Adequacy of follow-up** | **Score** | **Rating** |
| De Souza *et al.* | 2020 | * |  | * |  | * | * | * | 5 | Medium |
| Lozano *et al.* | 2018 | * |  | * |  | * | * | * | 5 | Medium |

Selection: Maximum of one star for each criteria

1. Case description 🡪 one star given if the study population is clear and fully described
2. Sample size🡪 was the number of cases adequate (n=10)
3. Ascertainment of exposure 🡪 one star given if validation methods are described sufficiently

Comparability: Maximum of one star for each criteria

1. Comparability on cohort on the basis of design:

- One star given if all patients across cohorts are CF patients

Outcomes: Maximum of one star for each category

1. Defined outcomes 🡪 one star given if all the outcomes measures are clearly defined
2. Sufficient follow-up 🡪 One star given if each patient is tracked for one year or more
3. Adequacy of follow-up of cohorts 🡪 One star given if complete follow-up with all patients or subjects lost to follow-up unlikely to introduce bias (under 10% lost)

Rating of quality of evidence:

- High = 6-7
- Medium = 4-5
- Low = 0-3

**Table S8: JBI Risk of Bias Assessment tool for prevalence studies (n=18)**

| Study | Year | Sample Representativeness | Appropriate Sampling | Adequate Sample Size | Sufficient study participant description | Sufficient coverage of identified sample | Valid methods used for identification? | Consistent measuring for all participants? | Appropriate statistical analysis | Adequate response rate? | Score | Rating |
| --- | --- | --- | --- | --- | --- | --- | --- | --- | --- | --- | --- | --- |
| Besier *et al.* | 2007 | ** | * | * | *** | - | * | * | * | * | 11 | High |
| Carzino *et al.* | 2017 | * | * | - | *** | * | * | - | * | * | 9 | Moderate |
| Dodement *et al.* | 2019 | ** | * | * | * | - | - | * | * | - | 7 | Moderate |
| Haussler *et al.* | 2003 | - | - | * | ** | * | * | - | - | - | 5 | Low |
| Kahl *et al.* | 1998 | * | - | - | ** | - | * | - | * | * | 6 | Moderate |
| Kahl *et al.* | 2003 | * | * | - | * | - | * | - | * | * | 6 | Moderate |
| Masoud-Landgraf *et al.* | 2016 | ** | * | - | - | - | * | * | * | * | 7 | Moderate |
| Moisan *et al.* | 2005 | - | - | - | - | - | * | - | * | * | 3 | Low |
| Morelli *et al.* | 2015 | * | * | - | *** | - | * | - | * | * | 8 | Moderate |
| Pakasticali *et al.* | 2016 | * | - | - | ** | - | * | - | * | * | 6 | Moderate |
| Schneider *et al.* | 2008 | ** | * | - | *** | - | * | * | * | * | 10 | High |
| Schwerdt *et al.* | 2018 | ** | * | * | - | - | * | * | * | * | 8 | Moderate |
| Suwantarat *et al.* | 2018 | * | * | * | - | - | * | - | * | * | 6 | Moderate |
| Tkadlec *et al.* | 2015 | ** | - | - | ** | - | * | - | * | * | 7 | Moderate |
| Vergison *et al.* | 2007 | * | * | * | * | - | * | * | * | - | 7 | Moderate |
| Wolter *et al.* | 2013 | * | * | - | *** | * | * | * | * | * | 10 | High |
| Wolter *et al.* | 2019 | * | * | - | *** | * | * | * | * | * | 10 | High |
| Yagci *et al.* | 2011 | ** | - | * | *** | - | * | - | * | * | 9 | Moderate |

Assessment Criteria:

1. Sample representativeness 🡪 Two stars given if the patient cohort is truly representative of the average CF patient. One star given in there are no age/sex/ethnicity restrictions, and another star given if there are no exclusions of patients depending on their colonization status, i.e. patients are excluded if they are colonized with *P. aeruginosa*
2. Appropriate sampling 🡪 One star given if the recruitment of participants is clearly described and selection of participants for inclusion is not hinted. E.g. It is stated that all respiratory specimens acquired by a CF centre were screened instead of a random amount
3. Adequate sample size 🡪 One star given if sample size is over 245. This ideal sample size was determined by the methods described by Cochrane (1)
4. Sufficient patient description 🡪 One star given for each of the following SCV population descriptions - age specifications, sex proportions and prior antibiotic use
5. Sufficient coverage of samples 🡪 One star given if sex proportions and adult vs paediatric classifications in the SCV group are identified
6. Valid identification methods 🡪 One star given if method of identification is described sufficiently
7. Consistent measuring across participants 🡪 One star given if the method of measuring and timing of measuring was consistent across all participants and is justified
8. Appropriate statistical analysis 🡪 One star given if statistical tests are clearly described and appropriate
9. Adequate response rate 🡪 One star given if complete follow-up with all patients or subjects lost to follow up is unlikely to introduce bias (under 10% lost)

Rating of quality of evidence:

- High: 10-12
- Moderate: 6-9
- Low: 0-5


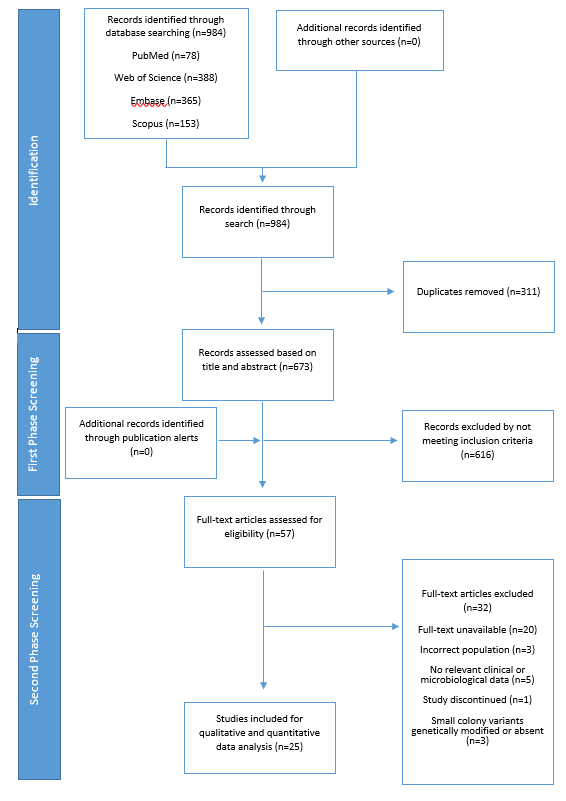


**Figure S1: Literature flow diagram of the search procedure.**

**Figure S2:** Funnel plot of the reported prevalence of SCVs in included studies. Dotted line represents pseudo 95% confidence limits. ES, Estimate size (Prevalence as a proportion); se(ES), standard error.

**Figure S3:** Sensitivity analysis: Forest plot of the prevalence of SCVs in Gram-positive studies (n=15 minus Schwerdt *et al.* 2018) utilising a random effects model with the DerSimonian and Laird method. Diamond represents estimated prevalence. ES, Estimate Size

**Figure S4:** Sensitivity analysis: Funnel plot of the reported prevalence of SCVs in included studies (n=13). Dotted line represents pseudo 95% confidence limits. SE, standard error; ES, estimate size.

**Figure S5:** Funnel plot of the reported mean difference of FEV_1_% between SCV and NCV participants in included studies (n=6). Dotted lines represents pseudo 95% confidence limits. WMD, Weighted mean difference; se(ES), standard error (WMD).

**Figure S6:** Sensitivity analysis: Forest plot of the reported mean difference of FEV_1_% between SCV and NCV participants in included studies (n=5 minus Wolter *et al.* 2019)) utilising a random effects model with the DerSimonian and Laird method. Diamond represents weighted mean difference between SCV and NCV participants. WMD, weighted mean difference.

**Figure S7:** Sensitivity analysis: Funnel plot of the reported mean difference in FEV_1_% between SCV and NCV participants in included studies (n=5 minus Wolter *et al.* 2019). Dotted lines represents pseudo 95% confidence limits. SE, standard error; WMD, weighted mean difference.

**Table S9: Specimens used to collect Gram-positive and Gram-negative respiratory pathogens in patients with CF**

| Sample types | Gram-positive | Gram-negative |
| --- | --- | --- |
|  | **Number of studies (%)** | **Number of studies (%)** |
| Blood culture | 2/18 (11.1) | 0/3 (0.0) |
| Respiratory Sample (Sputum) | 17/18 (94.4) | 2/3 (66.7) |
| Respiratory Sample (Endotracheal aspirate) | 1/18 (5.6) | 0/3 (0.0) |
| Respiratory Sample (Deep throat sample) | 12/18 (66.7) | 1/3 (33.3) |
| Bronchioalveolar lavage | 5/18 (27.8) | 0/3 (0.0) |
| Respiratory Sample (unspecified) | 1/18 (5.6) | 1/3 (33.3) |

**Table S10: Growth characteristics of SCVs of Gram-positive and Gram-negative respiratory pathogens.**

|  | Description | Number of studies (%) |
| --- | --- | --- |
| Gram-positive | Greyish/non-pigmented | 16/19 (84.2) |
|  | Slow-growing | 7/19 (36.8) |
|  | Small | 17/19 (89.5) |
|  | Non-haemolytic | 17/19 (89.5) |
|  | Fried egg morphotype | 5/19 (26.3) |
|  | Normal growth on Schaedler agar | 6/19 (31.6) |
| Gram-negative | Slow-growing | 1/1 (100.0) |
|  | Small | 2/2 (100.0) |
|  | Maintains small phenotype in at least two subcultures | 3/3 (100.0) |

**Table S11: Agar mediums used for cultivation of SCVs of Gram-positive and Gram-negative respiratory pathogens.**

|  | Description | Number of studies (Valid %) |
| --- | --- | --- |
| Gram-positive | Columbia sheep blood agar | 4/17 (23.5) |
|  | Blood agar | 10/17 (58.8) |
|  | Brucella agar | 3/17 (17.6) |
|  | Chocolate agar | 3/17 (17.6) |
|  | Mannitol salt agar | 11/17 (64.7) |
|  | Schaedler agar | 5/17 (29.4) |
| Gram-negative | Columbia sheep blood agar | 3/3 (100.0) |
|  | MacConkey agar | 3/3 (100.0) |
|  | Brucella agar | 1/1 (100.0) |
|  | Chocolate agar | 1/1 (100.0) |
|  | OFBL agar | 1/1 (100.0) |

**Table S12: Incubation conditions used in cultivation of SCVs of Gram-positive and Gram-negative respiratory pathogens.**

|  | Gram-positive | Gram-negative |
| --- | --- | --- |
|  | **Number of studies (%)** | **Number of studies (%)** |
| *Incubation Time* |  |  |
| 24 hours | 11/16 (68.8) | 0/0 (0.0) |
| 48 hours | 10/16 (62.5) | 4/4 (100.0) |
| Over 72 hours | 1/16 (6.3) | 0/0 (0.0) |
|  |  |  |
| *Incubation Conditions* |  |  |
| 35°C | 11/16 (68.8) | 2/3 (66.7) |
| 37°C | 1/16 (6.3) | 2/3 (66.7) |
| 5-10% CO_2_ | 5/16 (31.3) | 0/0 (0.0) |

**Table S13: Tests used for the confirmation of SCVs of Gram-positive and Gram-negative respiratory pathogens.**

|  | Follow-up test | Number of studies (Valid %) |
| --- | --- | --- |
| Gram-positive | 16S rRNA gene amplification | 4/18 (22.2) |
|  | PCR amplification of *nucA* | 8/18 (44.4) |
|  | PCR amplification of *mecA* | 5/18 (27.8) |
|  | Agglutination testing with Slidex Staph Plus | 10/18 (55.6) |
|  | Tube coagulase testing | 11/18 (61.1) |
|  | Catalase testing | 6/18 (33.3) |
| Gram-negative | Maltose oxidation | 1/1 (100.0) |
|  | Oxidase reaction testing | 1/1 (100.0) |
|  | Gas chromatography | 1/2 (50.0) |
|  | Colony morphology | 1/2 (50.0) |
|  | Growth at 42°C | 1/2 (50.0) |
|  | Characteristic pigment production | 1/2 (50.0) |
|  | Lysine decarboxylation | 1/1 (100.0) |
|  | Triple-sugar-iron agar slants | 1/1 (100.0) |
